# 1947. Activity of Rezafungin against Echinocandin–Non-wild type C. glabrata Clinical Isolates from the Rezafungin Surveillance Program (2014–2021)

**DOI:** 10.1093/ofid/ofad500.101

**Published:** 2023-11-27

**Authors:** Cecilia G Carvalhaes, Paul Rhomberg, Abby Klauer, Lalitagauri M Deshpande, Mariana Castanheira

**Affiliations:** JMI Laboratories, North Liberty, IA; JMI Laboratories, North Liberty, IA; JMI Laboratories, North Liberty, IA; JMI Laboratories, North Liberty, IA; JMI Laboratories, North Liberty, IA

## Abstract

**Background:**

Fluconazole (FLC) resistance (R) is common in *C. glabrata* (CGLA). Echinocandins (ECHs) are often used as first-line therapy. R to ECHs has been associated with FKS1 and FKS2 gene alterations. Rezafungin (RZF), a new ECH approved by the US FDA to treat candidemia and invasive candidiasis, was evaluated against a collection of ECH**–**non-wild type (NWT) CGLA isolates.

**Figure:**
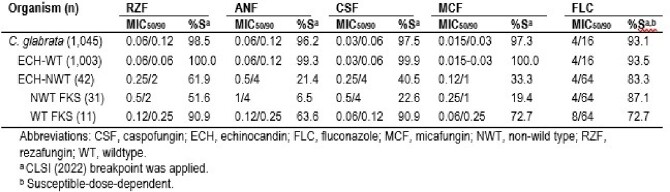

**Methods:**

A total of 1045 CGLA collected (1/patient) in 2014–2021 from 58 medical centers located in North America (NA; *n*=459; 20 centers), Europe (EU; *n*=396; 23 centers), Asia-Pacific (AP; *n*=130; 10 centers), and Latin America (LA, *n*=60; 5 centers) were identified by MALDI-TOF and/or sequencing and tested by CLSI broth microdilution. CLSI breakpoints (BP) and epidemiological cut-off values were applied. ECH NWT isolates were submitted to *FKS* analysis by whole genome sequencing.

**Results:**

RZF showed similar activity to other ECHs against CGLA (Table), inhibiting 98.5% at ≤0.5 mg/L. Anidulafungin (ANF), caspofungin (CSF), and micafungin (MCF) susceptibility (S) rates were 96.2%, 97.5%, and 97.3%, respectively. ECH-NWT CGLA were detected in 42 isolates (4.0% overall): NA showed the highest rate of ECH NWT isolates (29; 6.3%), followed by EU (10; 2.5%), AP (2; 1.5%), and LA (1; 1.7%). The RZF S rate was 61.9% of ECH-NWT CGLA, while the S rate to ANF, CSF, and MCF was 21.4%, 40.5%, and 33.3%, respectively. Alteration in *FKS* genes were observed in 31 CGLA (73.8% of ECH NWT; 3.0% overall). RZF was active against >50% of these isolates. RFZ was also active against >90% of ECH-NWT CGLA carrying wildtype *FKS* genes. Equivalent activity was noted to other ECHs against ECH-NWT isolates. However, ECHs were 4 to 8-fold more active against ECH-NWT CGLA that did not display alterations in *FKS* genes than those with *FKS* alterations. FKS2 hot spot(HS)-1 alterations were observed in 22 isolates (12 displayed S663F), while FKS1-HS1 alterations were noted in 9 isolates (7 displayed S629P). FLC-R was observed in 6.9% of CGLA overall and 16.7% of the ECH-NWT isolates.

**Conclusion:**

RZF demonstrated potent *in vitro* activity against CGLA and remained active against most isolates of CGLA displaying an ECH-NWT phenotype with or without *FKS* alterations.

**Disclosures:**

**Cecilia G. Carvalhaes, MD, PhD**, AbbVie: Grant/Research Support|bioMerieux: Grant/Research Support|Cipla: Grant/Research Support|CorMedix: Grant/Research Support|Melinta: Grant/Research Support|Pfizer: Grant/Research Support **Paul Rhomberg, BS, MT(ASCP)**, bioMerieux: Grant/Research Support|Melinta: Grant/Research Support|Pfizer: Grant/Research Support **Abby Klauer, BS**, Melinta: Grant/Research Support **Lalitagauri M. Deshpande, PhD**, Melinta: Grant/Research Support|Paratek: Grant/Research Support **Mariana Castanheira, PhD**, AbbVie: Grant/Research Support|Basilea: Grant/Research Support|bioMerieux: Grant/Research Support|Cipla: Grant/Research Support|CorMedix: Grant/Research Support|Entasis: Grant/Research Support|Melinta: Grant/Research Support|Paratek: Grant/Research Support|Pfizer: Grant/Research Support|Shionogi: Grant/Research Support

